# Distribution of Thyroid Cancer in the Eastern Part of Turkey 27 Years After the Chernobyl Accident

**DOI:** 10.4021/wjon726w

**Published:** 2014-01-16

**Authors:** Serap Baydur Sahin, Ahmet Fikret Yucel, Hasan Gucer, Ahmet Pergel, Recep Bedir, Ibrahim Aydin, Ibrahim Sehitoglu, Dursun Ali Sahin, Osman Zikrullah Sahin

**Affiliations:** a Department of Endocrinology and Metabolism Disease, Recep Tayyip Erdogan University, Rize, Turkey; b Department of Surgery, Recep Tayyip Erdogan University, Rize, Turkey; c Department of Pathology, Recep Tayyip Erdogan University, Rize, Turkey; d Department of Nephrology, Recep Tayyip Erdogan University, Rize, Turkey

**Keywords:** Thyroid cancer, Distribution, Chernobyl accident

## Abstract

**Background:**

The Chernobyl accident caused widespread effects across Europe and huge areas where radiocontaminated. The effects of the Chernobyl accident on thyroid cancer have been investigated in most European countries. According to the data of the Turkish Atomic Energy Authority, the eastern part of the Black Sea region was the most radiocontaminated area in Turkey at the time of Chernobyl accident. We therefore aimed to examine the data of thyroid cancers at our center, Rize city which is located in the eastern Black Sea region.

**Methods:**

This retrospective study included the patients with histologically proven thyroid cancer at our center between January 2008 and May 2012. Pathologic examinations of thyroidectomy materials were reviewed. We evaluated patients’ age, gender, size of the primary tumor (all sizes, < 1 cm, 1 - 2.9 cm, 3 - 3.9 cm and ≥ 4 cm), multicentricity, histologic subtypes of thyroid cancer, the presence of lymphatic, vascular, capsule and the extrathyroidal invasion.

**Results:**

Five hundred and forty-seven of the 3,556 patients were diagnosed with thyroid cancer. The mean age of the patients was 49.31 ± 0.49 years. The histopathologic diagnosis of patients was papillary carcinoma in 533 (97.4%) and the tumor size was < 1 cm in 53.6% of the patients. The presence of multicentricity was detected in 47% of the patients.

**Conclusion:**

The portion of thyroid carcinomas in all thyroidectomies was 15.4% in our institution 27 years after the Chernobyl accident.

## Introduction

Chernobyl nuclear power plant accident happened on April 26, 1986 and led to release of huge amounts of radioactive isotopes. Many millions of people in Belarus, northern Ukraine and adjacent parts of the Russian Federation were exposed to significant fallout. Eight days after the accident, the radiation cloud was still present over Europe and low amounts of radioactivity were detected in large parts of Europe, such as Austria, Italy and France [1]. Turkey is closer to the place of the accident than most European countries, but fortunately the disaster did not affect the whole country except the north eastern part [2]. Rize, province of the eastern part of Black Sea is synonymous with tea production in Turkey. It is known that fields growing up tea received rainfall when the radiation cloud was passing 12 - 14 days after the Chernobyl nuclear meltdown [2].

After radioactive fallout from Chernobyl, the largest human exposure was to Iodine 131, so that the thyroid received much larger doses than the other tissues. Beginning in 1990, 4 years after the accident, a sudden increase in the numbers of thyroid cancer occurring in the children in the most affected areas was noted [3-9]. Since then, numerous studies have demonstrated a relationship between I-131 exposure from Chernobyl and thyroid cancer risk [10-12]. The thyroid cancer risk is related to the distance from the explosion and inversely related to the age of those exposed [13, 14]. It is unknown whether radiation from the Chernobyl accident increased the incidence of thyroid cancer in those who were adults at the time of the accident. Latency times between radiation exposure and development of thyroid cancer range from a minimum of 3 - 7 years to a maximum of 40 - 50 years.

There are not enough data whether Chernobyl disaster has an effect on development of thyroid carcinomas (TCs) in Turkey. The eastern part of the Black Sea region was the most radiocontaminated area in Turkey at the time of the Chernobyl accident [2]. We therefore aimed to examine the data of thyroid cancers at our center, Rize.

## Materials and Methods

This retrospective study included all consecutive patients who underwent total thyroidectomy (TT) between January 2008 and May 2012. Most of the thyroidectomies were performed in our hospital. The other patients were operated in the other four hospitals in Rize region. All the specimens were evaluated in the Pathology Department of our hospital. Pathologic examinations of thyroidectomy materials were reviewed. Five hundred and forty-seven patients were diagnosed with thyroid cancer.

The patients with histologically proven thyroid cancer were examined and the following information was recorded: patients’ age, gender, size of the primary tumor (all sizes, < 1 cm, 1 - 2.9 cm, 3 - 3.9 cm and ≥ 4 cm), multicentricity, histologic subtypes of thyroid cancer, the presence of capsule, lymphatic, vascular and the extrathyroidal invasion.

### Statistical analysis

Demographical and histopathologic data were recorded retrospectively, using SPSS version 14.0 software (SPSS Graduate Pack 14.0; SPSS Inc., Chicago, IL, USA). Chi-square test or the Mann-Whitney U test was used for statistical analysis. With regard to the results, statistical significance was defined as P < 0.05.

## Results

Between January 2008 and May 2012, 3,661 patients with thyroid disease underwent TT. Five malignancies in the thyroid gland were excluded (1 non-Hodgkin lymphoma, 1 metastasis of a clear cell renal carcinoma, 2 unclassified differentiated cancer and 1 squamoz cell ca).

Five hundred and forty-seven of the 3,556 patients (15.4%) were diagnosed with thyroid cancer and these patients consisted of 453 (82.8%) women and 94 men (17.2%) with an age range between 17 and 81 (mean: 49.31 ± 0.49) (Table 1).

**Table 1:** The Characteristics and Histopathologic Diagnoses of Patients

Parameter	
Age (years)	49.31 ± 0.49
The proportion of patients under 18 years at exposure	29.6%
Gender (female %)	82.8%
Papillary carcinoma (%)	97.4%
Follicular carcinoma (%)	1.4%
Medullary carcinoma (%)	0.7%
Anaplastic/undifferentiated carcinoma (%)	0.5%

The histopathologic diagnoses of patients were papillary carcinoma in 533 (97.4%), follicular carcinoma in 8, medullary carcinoma in 4 and anaplastic/undifferentiated carcinoma in 2 patients. The subtypes of papillary carcinoma included 340 (63.8%) classic papillary, 145 (27.2%) follicular variant, 40 (7.5%) oncocytic variant and 8 (1.5%) others (tall cell, diffuse sclerosan and columnar cell).

Mean size of the primary tumor was 12.87 ± 0.57 mm. With regard to tumor size, the ratios of patients are presented in Table 2. Of 547 patients in whom thyroid carcinoma was diagnosed, 150 (27.4%) had non-specific diffuse chronic lymphocytic thyroiditis. The pathologic features of the patients are summarized in Table 3.

**Table 2:** Tumor Characteristics of the Patients With Regard to Tumor Size

Tumor size	Patients (%)
< 1 cm	293 (53.6%)
1 - 2.9 cm	190 (34.7%)
3 - 3.9 cm	28 (5.1%)
≥ 4 cm	36 (6.6%)

**Table 3:** Pathologic Features of the Patients With Thyroid Carcinoma

Pathologic features	Without	With
Multicentricity (%)	290 (53%)	257 (47%)
Tumor capsule invasion (%)	442 (80.8%)	105 (19.2%)
Lymphatic invasion (%)	522 (95.4%)	25 (4.6%)
Vascular invasion (%)	521 (95.2%)	26 (4.8%)
Extrathyroidal invasion (%)	503 (92%)	44 (8%)

## Discussion

In the current study, we evaluated the thyroid cancer distribution who underwent TT in Rize, the most radiocontaminated area in Turkey at the time of the Chernobyl accident. According to the data of the Turkish Atomic Energy Authority, average radiocaesium contamination was found at 900 Bq/m^2^, and average radioiodine contamination was found to be as high as 8,000 Bq/m^2^ in Turkey just after the Chernobyl accident. The estimated radioactivity per person in the Turkish population was 1,100 Bq [2]. After the Chernobyl accident, in a study investigating the Cs134 and Cs137 radyonuclids in lichens demonstrated that the eastern part of Black Sea was the mostly affected region in Turkey [15]. In our study, 15.4% of the patients who were operated were diagnosed with thyroid cancer in Rize.

A number of studies estimated thyroid cancer risk of people exposed to Chernobyl radiation in childhood and adolescence, in Ukraine, Russia and Belarus and have demonstrated significantly an increased radiation risk [16-18]. After the accident, from 1989 to 2007, the thyroid cancer incidence rate increased from 0.9 to 1.6 in males and from 2.7 to 6.2 in females, per 100,000 individuals in Ukraine [19]. However, the exact impact of the Chernobyl accident on thyroidal cancer development in Europe was difficult to define. Some of the studies did not favor any link between the increased incidence of thyroid cancers and the Chernobyl accident in western European countries [20-22]. However, several studies in Europe, suggested a link with the Chernobyl accident, but it was thought that factors including improvements in ascertainment and earlier detection of tumors may also have contributed to the increasing incidence [10-12]. The data from the National Cancer Institute’s Surveillance, Epidemiology, and End Results (SEER) program showed that the incidence of thyroid cancer increased from 3.6 per 100,000 in 1973 to 8.7 per 100,000 in 2002, a 2.4-fold increase in the United States and 87% of the increase was due to the small papillary cancers [23]. In a study from Luxembourg, it was demonstrated that the incidence rates of thyroid carcinoma over the two 5-year periods 1990-1994 and 1995-1999 increased from 7.4 per 100,000 to 10.1 per 100,000 in females, from 2.3 per 100,000 to 3.6 per 100,000 in males and this increase was mainly due to the rise in the number of microcarcinomas [24].

According to the Turkish Cancer Research Association in 2008, the thyroid cancer incidence rate increased from 1 to 3.5 in males and from 3.6 to 15.3 in females, per 100,000 individuals in Turkey [25]. However, Zengi et al did not demonstrate an increase in thyroid papillary cancer from 1982 to 2006 in the Agean region of Turkey [26]. In another study from Turkey, thyroid cancer was detected in 12.6% of patients who underwent thyroidectomy in years between 1999 and 2007 and this result is similar with our findings [27].

Exposure to external radiation [28] or to fallout from nuclear weapon testing, genetic predisposition [29], residence in an endemic goiter area, or history of thyroid disease [28] and parity [30] may have etiologic implications. Recently, changes in practices for the management of thyroid diseases are considered to be a main potential etiologic factor of increased incidence [23, 31-34]. However in a study, the increasing incidence of differentiated thyroid cancer in all sizes suggested that increased diagnostic scrutiny is not the sole explanation [34]. In our study, 53.6% of the tumors were measured < 1 cm, so this may be the result of more frequent use of ultrasonography in locating thyroid nodules and the subsequent use of fine-needle aspiration to ascertain pathology. Ultrasound-guided fine-needle aspiration of non-palpable thyroid nodules contributed to the increasing incidence of early thyroid cancer.

In our study, papillary carcinoma was the most common (97.4%) histologic subtype. The relationship between the increase in the incidence of papillary cancer and iodine supplementation has been demonstrated in several studies [35, 36]. Turkey is a region of endemic goitre and legislation for mandatory iodization of household salt was passed in July 1999 [37]. Thus, the high ratio of papillary to follicular carcinoma may be due to iodine supplementation.

The age at exposure has been shown to be an important factor in the risk of developing TC after the fallout from Chernobyl, especially under the age of 1, which has shown a very much greater risk than older children at exposure [1]. In our study, the mean age of patients was 49.3. Only 29.6% of the patients were under 18 years at exposure. When we evaluated the tumor size according to the patients’ age < 18 years and ≥ 18 years at exposure, we did not find a significant difference (P = 0.707). Additionally, the pathologic features were similar in the two groups.

Chernobyl-related papillary TCs have been shown to be associated with somatic mutations such as RET/PTC rearrangements [38]. Unfortunately, molecular tests could not be performed in our study. The other limitation for the present study is that we do not have enough data about the prevalence of thyroid cancer before 2008, so we cannot estimate whether there is an increase in the prevalence of thyroid cancer in our population.

In conclusion, 27 years after the accident, the portion of TCs in all thyroidectomy specimen was 15.4% in our institution. It is impossible to decide whether there is an impact of the Chernobyl accident on the prevalence and incidence of TC in eastern part of Black Sea region in Turkey.
